# Cost-Effective Transcriptome-Wide Profiling of Circular RNAs by the Improved-tdMDA-NGS Method

**DOI:** 10.3389/fmolb.2022.886366

**Published:** 2022-05-13

**Authors:** Ashirbad Guria, Priyanka Sharma, Nagesh Srikakulam, Akhil Baby, Sankar Natesan, Gopal Pandi

**Affiliations:** ^1^ Department of Plant Biotechnology, School of Biotechnology, Madurai Kamaraj University, Madurai, India; ^2^ Department of Genetic Engineering, School of Biotechnology, Madurai Kamaraj University, Madurai, India

**Keywords:** circRNA, i-tdMDA-NGS, circRNA sequencing, phi29 DNA polymerase, divergent PCR

## Abstract

Covalently closed circular RNAs are neoteric to the eukaryotic family of long non-coding RNAs emerging as a result of 5′–3′ backsplicing from exonic, intronic, or intergenic regions spanning the parental gene. Owing to their unique structure and stability, circular RNAs have a multitude of functional properties such as micro-RNA and protein sponges, direct and indirect modulators of gene expression, protein translation, and many unproven activities apart from being potential biomarkers. However, due to their low abundance, most of the global circular RNA identification is carried out by high-throughput NGS-based approaches requiring millions of sequencing reads. This lag in methodological advancements demands for newer, more refined, and efficient identification techniques. Here, we aim to show an improved version of our previously reported template-dependent multiple displacement amplification (tdMDA)-NGS method by superimposing the ribosomal depletion step and use of H minus reverse transcriptase and RNase H. Implication of tdMDA using highly replicative Phi29 DNA polymerase after minimizing the linear and ribosomal RNA content further intensifies its detection limit toward even the abysmally expressing circular RNA at a low NGS depth, thereby decreasing the cost of identifying a single circular RNA. A >11-fold and >6-fold increase in total circular RNA was identified from the improved-tdMDA-NGS method over the traditional method of circRNA sequencing using DCC and CIRI2 pipelines, respectively, from *Oryza sativa* subsp. *Indica.* Furthermore, the reliability of the improved-tdMDA-NGS method was also asserted in HeLa cell lines, showing a significant fold difference in comparison with the existing traditional method of circRNA sequencing. Among the identified circular RNAs, a significant percentage from both rice (∼58%) and HeLa cell lines (∼84%) is found to be matched with the previously reported circular RNAs, suggesting that the improved-tdMDA-NGS method can be adapted to detect and characterize the circular RNAs from different biological systems.

## Introduction

The circularization of the RNA molecules has been in the limelight for many decades (Sanger et al., 1976; Kos et al., 1986; Nigro et al., 1991; Capel et al., 1993; Houseley et al., 2006), while its potential was underestimated in various avenues of molecular bioprocesses in both animal and plant systems. The nature of their continuous loop structure as a result of backsplicing of the downstream 5′ donor and the upstream 3′ acceptor or from the lariat precursor (Chen and Yang, 2015; Cheng et al., 2016; Zhao et al., 2019) has given circular RNAs (circRNAs) a greater stability than their commonly expressed cognate linear RNA isoforms (Jeck and Sharpless 2014; Guria et al., 2020; Rochow et al., 2020). With the technical advancements in detecting both canonical and non-canonical backsplice junctions (BSJs), these ubiquitously expressed circRNA molecules (Wang et al., 2014; Santer et al., 2019; Zhou et al., 2020) ranging from ∼100 nucleotides (nt) to >4 kilobases (kb) have been identified to span the exonic, intronic, and intergenic sequences throughout the genome in various combinations (Lasda and Parker, 2014; Guria et al., 2019; Jamal et al., 2019; Xin et al., 2021). The unique properties of circRNAs impart significant functional capabilities such as therapeutic biomarkers (Liang et al., 2021; Sarkar and Diermeier, 2021; Tian et al., 2021), micro-RNA decoy (Yu and Kuo, 2019; Olesen and Kristensen, 2021; Zeng et al., 2021), RBP sponging (Zang et al., 2018; Okholm et al., 2020; Das et al., 2021), and putative cap-independent protein translation (Legnini et al., 2017; Yang et al., 2017; Lei et al., 2020; He et al., 2021) in order to modulate and regulate many biological processes and pathologies.

CircRNAs are 1–3% of the total poly(A) RNA population in the transcriptomic pool (Salzman et al., 2013), and identification of these extremely low abundant molecules remains a challenge and an expensive occurrence to the traditional method of rRNA-depleted circRNA sequencing. In order to overcome these limitations, for the first time, we, in our previous report (Guria et al., 2019), have harnessed the potential of phi29 DNA polymerase in the field of circRNA identification. However, due to the low amount of template concentration, multiple displacement amplification (MDA) is prone to template-independent amplification (TIA) when a high concentration of a random hexamer is used at an extended incubation period (Wang W. et al., 2017; Guria et al., 2019). Although the mechanism of TIA remains unclear, it is thought to be triggered as a result of Phi29 DNA polymerase jumping and self-priming of the random oligonucleotides, which eventually compromises the MDA outcome as a result of the undesirable amplicon product (Wang Y. et al., 2017). Since we adopted MDA to identify circRNAs, we selected the exo-resistant random pentamer (ERRP) (/5Sp18/NNN*N*N) in order to inhibit self-priming and the template switching property of Phi29 DNA polymerase (Wang W. et al., 2017; Guria et al., 2019). We hypothesized from the earlier report (Guria et al., 2019) that depleting the highly structured ribosomal RNAs (rRNAs) would enrich the circRNAs. Subjecting these enriched circRNAs to RNase H minus RT would result in long-length cDNAs that would eventually enhance the sensitivity of phi29 DNA polymerase-mediated MDA to efficiently capture even the poorly expressed circRNAs, thereby increasing the circRNA detection capacity. Therefore, currently, we optimized the phi29 DNA polymerase-mediated template-dependent MDA (tdMDA) to enrich the overall putative circRNAs by implementing the rRNA depletion step followed by RNase R treatment to enhance its sensitivity toward the linear RNAs. This approach yields 70,908 and 34,213 circRNAs from rice and HeLa cell lines, respectively, using DCC (Cheng et al., 2016) from as low as ∼30 million paired end reads as opposed to 6,368 and 8,958 circRNAs using the conventional method of circRNA sequencing which does not involve tdMDA. Further, the number of identified circRNAs is reduced to 2,517 and 710 from rice and HeLa cell lines, respectively, in i-tdMDA-NGS using CIRI2 pipelines (Gao et al., 2018), whereas traditional RNA-Seq yields only 408 and 8,499 circRNAs from rice and HeLa cell lines, respectively. Thus, developing i-tdMDA-NGS is found to be an efficient and equally effective methodology compared to traditional RNA-Seq when balanced with appropriate computational algorithms. It is highly sensitive in identifying the low-expressing circRNAs, having canonical or non-canonical backsplice junctions and different circRNA types from a small amount of sample and being cost effective at the same time.

## Materials and Methods

### Sample Collection and RNA Isolation

*Oryza sativa* subsp. *Indica* was grown and maintained in a green house under controlled conditions of a 32°C temperature, 70–80% humidity, and a 16/8 hours day–night cycle. Old leaf materials (35–42 days, 300 mg) were collected and ground to fine powder using liquid nitrogen in a pre-chilled mortar–pestle. RNA was extracted using RNAiso Plus (Takara Bio, Kusatsu, Shiga, Japan) reagent by following the method as mentioned previously (Guria et al., 2019).

HeLa cells were obtained from NCCS Pune, India, and were maintained under an atmosphere of 37°C, 5% CO_2_ in RPMI-1640 media (with L-Glutamine, Gibco, Thermo Fisher Scientific, Waltham, MA, United States) supplemented with 10% fetal bovine serum (Gibco, Thermo Fisher Scientific, Waltham, MA, United States) and 1% penicillin–streptomycin (HiMedia, Mumbai, Maharashtra, India) antibiotics. The cells were harvested in 1 ml of RNAiso Plus (Takara Bio, Kusatsu, Shiga, Japan) using a cell scrapper upon reaching 70–80% confluent state. The collected HeLa cells were passed four to five times through a 1 ml syringe in order to lyse the cells, and total RNA was isolated using RNAiso Plus (Takara Bio, Kusatsu, Shiga, Japan) using the manufacturer’s protocol.

### Enrichment of circRNA and its Reverse Transcription

For every 10 μg of total RNA extracted from both *O. sativa* subsp. *Indica* and HeLa cells, 4 U Turbo DNase (2 U/μl, Ambion, Austin, Texas, United States) was used at 37°C for 30–45 min. The reaction was heat-inactivated using 0.01 mM ethylenediaminetetraacetic acid for 10 min at 70–75°C, followed by phenol/chloroform purification. The quantity and quality of the RNA were assessed using NanoDrop ND-1000 spectrophotometer (Thermo Fisher Scientific, Waltham, MA, United States) readings, followed by running 1.5% MOPS agarose gel electrophoresis. After ensuring the RNA quality, 10 μg of DNA-depleted RNA was subjected to rRNA depletion using a RiboMinus™ Plant Kit for RNA-Seq (Thermo Fisher Scientific, Waltham, MA, United States) and RiboMinus™ Eukaryote Kit v2 (Thermo Fisher Scientific, Waltham, MA, United States) for rice and HeLa cells, respectively, by adhering to the manufacturer’s instructions. The rRNA-depleted RNA was subjected to RNase R (20 U/μl, Epicentre, United States) digestion at 37°C for 20 min.

The entire enriched RNA was reverse-transcribed with random primers and oligo-dT primers separately using a RevertAid H minus first strand cDNA synthesis kit according to the manufacturer’s instructions (Thermo Fisher Scientific, Waltham, MA, United States). The converted complementary DNA (cDNA) was treated with 0.5 μL of RNase H (5 U/μl, Thermo Fisher Scientific, Waltham, MA, United States) in 20 µL reaction at 37°C for 20 min, followed by enzyme inactivation at 65°C for 10 min.

### Abolishing Template-Independent Amplification by Phi29 DNA Polymerase

For eliminating template-independent amplification (TIA), circular pBluescript (pBKSII+) and water were used as a positive control and negative control, respectively, for phi29 DNA polymerase amplification. They were incubated with either an exo-resistant random hexamer (NNNN*N*N) (ERRH) having two phosphothioarate bonds at the 3′end or an exo-resistant random pentamer (/5Sp18/NNN*N*N) (ERRP) having the 5′end blocked by the C18 spacer apart from two phosphothioarate bonds at the 3′end at two different final concentrations (30 μM, 50 µM) at either 28°C or 30°C for 16, 18, or 21 h with other reagents and protocols as followed previously (Guria et al., 2019).

The ss cDNA produced (*Enrichment of circRNA and its Reverse Transcription Section*) from both rice and HeLa cells was used separately as a template for tdMDA by incubating with 30 µM exo-resistant random pentamer at 28°C for 21 h along with other reagents as mentioned in Guria et al. (2019) to obtain a high-molecular-weight amplicon.

### Library Preparation, Illumina Sequencing, and Bioinformatic Analysis of circRNA

The processed enriched circRNA (25–100 ng) (*Enrichment of circRNA and its Reverse Transcription Section*) and 250 ng of circRNA-derived td-MDA products (*Abolishing Template Independent Amplification (TIA) by Phi29 DNA Polymerase Section*) from both rice and HeLa cells were used for library preparation using an Illumina-compatible NEBNext^®^ Ultra™ II Directional RNA Library Prep Kit (New England BioLabs, MA, United States) and an Illumina-compatible NEXTflex Rapid DNA sequencing Bundle (BIOO Scientific, Inc., United States), respectively, as per the manufacturer’s instructions at Genotypic Technology Pvt. Ltd., Bangalore, India. The prepared sequencing libraries were purified with JetSeq beads (Bioline, Meridian Bioscience, Memphis, Tennessee, United States) and quantified using a Qubit fluorometer (Thermo Fisher Scientific, Waltham, MA, United States), and their fragment size distribution was analyzed on a 2200 TapeStation (Agilent, Santa Clara, CA, United States). They were sequenced in an Illumina platform [150 nt paired end (PE)] at Genotypic Technology Pvt. Ltd., Bangalore, India, as mentioned previously (Guria et al., 2019). The Raw Illumina sequence reads were submitted under Bioproject ID PRJNA803606 (SRA accession nos: SRR17967589, SRR17967587, SRR17967586, SRR17967585, SRR17967588 and SRR17967584) and PRJNA803607 (SRA accession nos: SRR17967603 and SRR17967602) for *O. sativa* subsp. *Indica* and HeLa cell lines respectively. The quality of the raw reads was checked by Phred25, and the AdapterRemoval V2 (Schubert et al., 2016) tool was employed to trim the adapter and low-quality sequences. The quality-filtered clean reads were used for both DCC (Cheng et al., 2016) and CIRI2 (Gao et al., 2018) analysis for circRNA identification. DCC analysis was performed in both paired-dependent and independent modes by considering the stranded and non-stranded parameters [DCC (paired-dependent, non-stranded): D -N -Nr 1 1 -G; DCC (paired-dependent, stranded): D -Nr 1 1 -G; DCC (paired-independent, non-stranded): D -N -Nr 1 1 -Pi -G; DCC (paired-independent, stranded): D -Nr 1 1 -Pi -G]. However, CIRI2 analysis was performed with zero stringency default settings as it provides no flexibility to alter its parameters like DCC software. As part of the DCC analysis, we have performed the STAR (Dobin et al., 2013) alignment according to the DCC manual and BWA-MEM (https://doi.org/10.48550/arXiv.1303.3997) alignment for the clean reads as per the CIRI2 manual. The processed reads from both rice and HeLa cells were aligned with their respective reference genome [ensembl plant release 29 (Howe et al., 2020) ASM475v1 for rice and the human hg38 genome (https://hgdownload.soe.ucsc.edu/downloads.html#human) for HeLa cells]. A minimum of one supporting read having the backsplice junction out of any one sample was considered for circRNA identification in both DCC and CIRI2 pipelines.

### Validation of Improved-tdMDA-NGS-Derived circRNAs

Randomly, two non-canonical circRNAs were selected and validated from a set of circRNAs under study by designing divergent primer pairs (Table 1). Divergent PCR was carried out using random primed cDNA and oligo-dT-derived cDNA for each circRNA at its optimized annealing temperature (T_A_). Further, the PCR products were gel-eluted by the silica matrix, cloned into pGEM-T Easy vector (Promega, Madison, WI, United States), and Sanger sequenced [Bioserve Biotechnologies (India) Pvt. Ltd., Secunderabad, India], for detection of the backsplice junction corresponding to each circRNA. qRT-PCR was also performed with two different cDNAs (*Enrichment of circRNA and its Reverse Transcription Section*) for checking the expression level of circRNAs.

**TABLE 1:** Table showing the list of divergent primers used in the study.

Organism	CircRNA	Primer	Sequence (5′-3′)	Length (nt)	Amplicon Size (bp)
Rice	Osi_circ_2-187437-187904_(-)	Osi_DC_02	AGA​AAG​GCA​TCG​ACG​ACA​TC	20	218
		FOR			
		Osi_DC_02	TGA​ACC​TGT​AGT​CGT​CGT​GC	20	—
		REV			
HeLa cell lines	Hl_circ_19-8963380-8964261_(-)	HL_DC_02	GTC​CCA​AGG​ATG​TGT​CCT​GG	20	267
		FOR			
		HL_DC_02	GAT​AAC​GCC​TCA​CCT​GCT​GT	20	—
		REV			

## Results

### Primer Optimization for Complete Elimination of Phi29 DNA Polymerase-Mediated TIA

At first, ERRH was used with the circular pBKSII+ plasmid (positive control) and no template (water control) at its designated final concentration of 50 µM and incubated with phi29 DNA polymerase at 30°C for 16 h. There was high-molecular-weight amplification with pBKSII+, but no band was seen in the water control, suggesting td-amplification (Supplementary Figure S1A). However, increasing to 21 h incubation yielded TIA (Supplementary Figure S1B), even when the primer concentration was reduced to 30 µM and incubated for 16 h (Supplementary Figure S1C). Later, the same positive and negative controls were taken with the exo-resistant random pentamer (ERRP) at 50 µM–16 h (Supplementary Figure S2A), 50 µM–21 h (Supplementary Figure S2B), and 30 µM–16 h (Supplementary Figure S2C) incubation at 30°C, which displays td-amplification. Minimizing the temperature to 28°C for ERRP produces tdMDA at 50 µM for 16, 18, and 21 h incubation (Supplementary Figures S2D–F). Finally, the ERRP concentration was reduced to 30 µM and when incubated at 28°C for 16, 18, and 21 h consistently shows no TIA (Supplementary Figures S2G–I). Hence, ERRP was optimized to a 30 µM final concentration and incubated at 28°C for 21 h to generate td-amplification for further downstream works.

### CircRNA Enrichment, NGS, and Read Analysis

**FIGURE 1:**
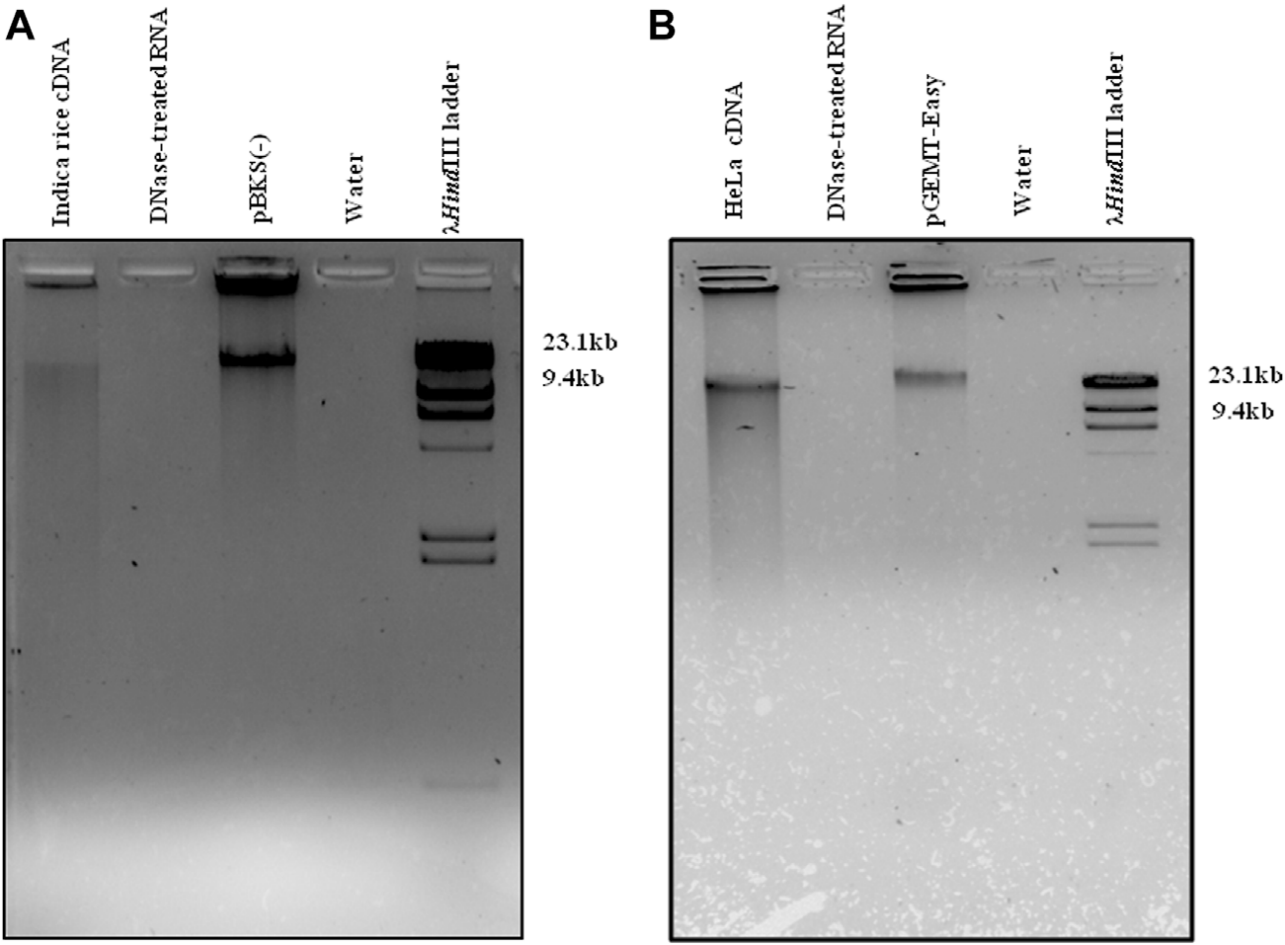
tdMDA in rice and HeLa. cDNA from **(A)** rice and **(B)** HeLa cell lines was incubated with phi29 DNA polymerase and 30 µM exo-resistant random pentamer at 28°C for 21 h, which displayed tdMDA along with the positive control (pBKS(-) and pGEMT-Easy), whereas DNase-treated RNA and water did not show any TIA. The λ*Hind*III (Thermo Fisher Scientific) ladder was loaded as a marker.

Total RNA was extracted from both *O. sativa* subsp. *Indica* (Supplementary Figure S3A) and HeLa cell lines (Supplementary Figure S3B), followed by removal of contaminating DNA using DNase from both samples (Supplementary Figure S3). The DNA-free RNA was hybridized against different 5′biotin labeled plant (nuclear-derived rRNAs-25/26S and 17/18S, mitochondrion-18S, and chloroplast-23S and 16S)- and animal (5, 5.8, 18, 28S)-specific rRNA probes (22–25 nt), which were pulled out by streptavidin-coated magnetic beads. The ribominus RNA was further treated with RNase R to eliminate all the remaining linear RNA populations resulting with leftover enriched circRNAs. One set of enriched circRNAs was processed for library preparation, followed by Illumina sequencing (traditional RNA-Seq), whereas the other set was converted into single-stranded (ss) linear cDNA using mutated (point mutation in the RNase H domain) Moloney Murine Leukemia Virus (M-MuLV) reverse transcriptase (RT), followed by RNase H treatment. The cDNA was used as a template for tdMDA under the optimized conditions using phi29 DNA polymerase and 30 µM ERRP at 28°C for 21 h (Figures 1A,B). The resulting linear double-stranded (ds) high-molecular DNA amplicon was used for library preparation, followed by NGS in an Illumina platform (improved (i)-tdMDA-NGS).

A total of 34,299,487 and 38,618,983 PE raw reads were obtained from biological triplicates of rice RNA (∼10 million PE reads/sample) using traditional RNA-Seq and i-tdMDA-NGS, respectively, which were processed at Phred25 to remove low-quality, adapter-specific, and other unwanted reads, resulting in retaining of 31,261,868 (91.14%) and 36,086,992 (93.44%) trimmed PE reads (Supplementary Figure S4). Similarly, 34,877,691 PE raw reads obtained from tdMDA-NGS in rice (Guria et al., 2019) were re-analyzed at the same Phred score to obtain 29,813,764 (85.48%) trimmed PE reads (Supplementary Figure S4), which were aligned against the reference genome, ASM465v1 (Ensembl plant, https://plants.ensembl.org/index.html).

On the other hand, 48,688,532 and 29,834,900 PE raw reads were trimmed at phred25 to get 48,599,461 (99.82%) and 28,803,581 (96.54%) PE reads (Supplementary Figure S5), respectively, from HeLa RNA using the traditional RNA-Seq and i-tdMDA-NGS methods, which were further aligned with the reference human hg38 genome (https://hgdownload.soe.ucsc.edu/downloads.html#human). The unaligned reads from both rice and HeLa cell lines were used in DCC and CIRI2 computational pipelines for circRNA identification.

### CircRNA Identification by CIRI2 and its Properties in Rice and HeLa Cell Lines

**FIGURE 2:**
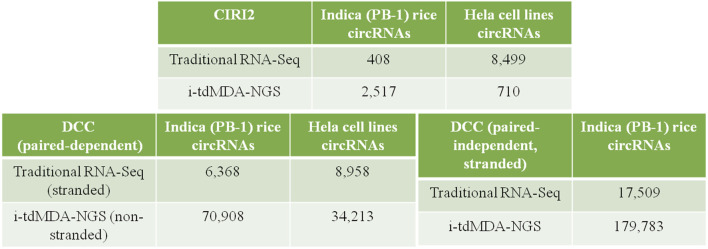
CircRNA identification by DCC and CIRI2. List showing circRNAs identified by traditional RNA-Seq and i-tdMDA-NGS using the DCC and CIRI2 computational pipelines from Indica rice and HeLa cell lines.

A total of 408 and 2,517 circRNAs were detected from the processed unaligned reads in rice by traditional RNA-Seq and i-tdMDA-NGS, respectively (Figure 2). Almost half of total circRNAs were arising from both positive and negative strands each (Supplementary Figure S6D) and distributed across 12 chromosomes in rice; nevertheless, almost 50% of circRNAs from both the methods were originated from chromosomes 1, 2, 3, and 4 (Supplementary Figure S6A), whereas >60 and 30% of total circRNAs were found to be exonic and intergenic circRNAs, respectively (Supplementary Figure S6B). ∼55% (#225) and ∼39% (#161) of circRNAs identified by traditional RNA-Seq were in the <1000 nt and 1000–9999 nt size range, respectively (Supplementary Figure S6C), whereas i-tdMDA-NGS contributes ∼45% (#1,143) and ∼51% (#1,299) of circRNAs in the same categories (Supplementary Figure S6C). In traditional RNA-Seq, 255 and 11 genes were giving single and two circRNAs, respectively, and 32% (#131) circRNAs were coming from genes without any annotation (Supplementary Figure S6E). On the contrary, there were 1,015 and 188 genes yielding one and two circRNAs, respectively, in i-tdMDA-NGS apart from ∼30% (#751) of circRNAs arising from unannotated genes (Supplementary Figure S6E).

Similarly, 8,499 and 710 circRNAs were identified in HeLa cell lines using traditional RNA-Seq and i-tdMDA-NGS, respectively (Figure 2), which were almost equally distributed in both the strands (Supplementary Figure S7D) in 22 chromosomes and chr X but not in chr Y and chr M (Supplementary Figure S7A). The maximum numbers of circRNAs were coming from chr 1, 2, either 5 or 3, and 12 from both the methods. About 83% (#7,057) of the circRNAs detected by traditional RNA-Seq were exonic types, followed by 13.8% (#1173) and 3.1% (#269) belonging to intergenic and intronic categories, respectively (Supplementary Figure S7B), unlike ∼50% of circRNAs each constituted by intergenic and intronic types when detected by i-tdMDA-NGS (Supplementary Figure S7B). A majority of circRNAs were large-sized of >1000 nt constituting ∼76% (#6,508) (1000–9999 nt - ∼49% + >10000 nt - ∼27%) and ∼90% (#636) (1000–9999 nt - ∼74% + >10,000 nt - ∼16%) of total circRNAs identified by traditional RNA-Seq and i-tdMDA-NGS, respectively (Supplementary Figure S7C). Finally, ∼26% (#2,194) and ∼40% (#289) of identified circRNA parental genes give rise to only single circRNA through the traditional RNA-Seq and i-tdMDA-NGS methodologies, respectively. However, ∼41% (#1,839) and ∼5% (#34) of genes produced >1 circRNAs besides #113 and #354 remaining unannotated circRNAs through traditional RNA-Seq and i-tdMDA-NGS, respectively. In addition to this, ∼2–5% of circRNAs identified through both the methods showed sequence overlapping with multiple genes (Supplementary Figure S7E).

### CircRNA Identification by DCC and its Properties in Rice and HeLa Cell Lines

**FIGURE 3:**
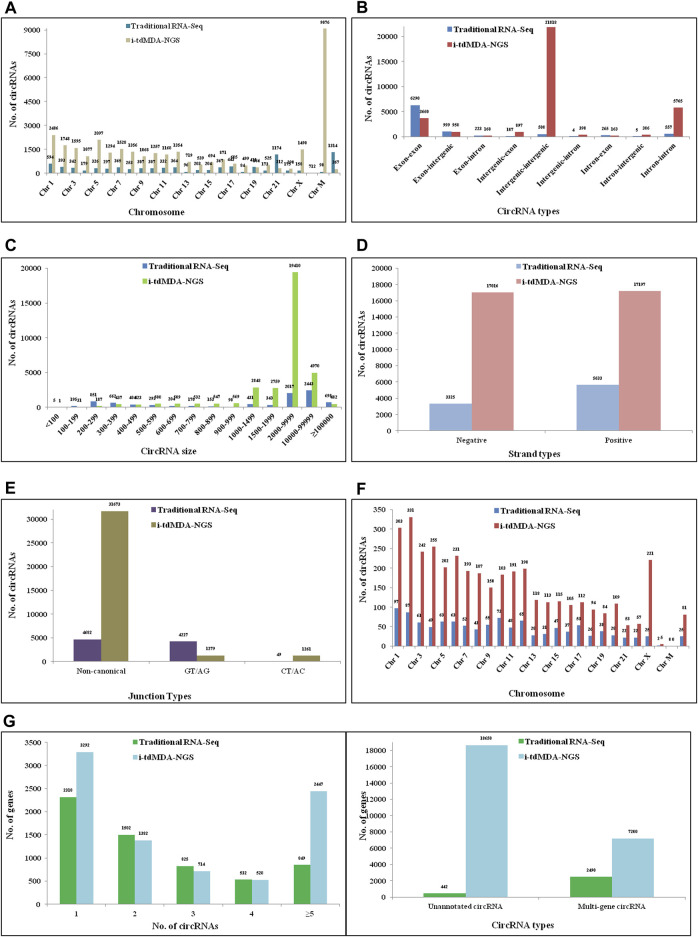
Comparative analysis of rice circRNAs by DCC. Rice circRNAs identified by traditional circRNA-Seq and i-tdMDA-NGS were analyzed by the DCC computational pipeline and compared based on its **(A)** location on the chromosome, **(B)** types, **(C)** size, **(D)** strand location, and **(E)** junction types; **(F)** expression of circRNAs over the corresponding linear RNA across chromosomes, and **(G)** number of circRNAs per host gene.

Using the DCC pipeline set in the paired-dependent mode, 6,368 and 70,908 circRNAs were identified from rice-processed reads (the same as used for CIRI2) using traditional RNA-Seq (analyzed in the stranded mode) and i-tdMDA-NGS (analyzed in the non-stranded mode), respectively (Figure 2), which were found to originate almost equally from both positive and negative strands (Figure 3D) and predominantly present on chromosome 1, 2, 3, and 5, thereby accounting for >40% of total identified circRNAs (Figure 3A). However, ∼20% of circRNAs from both the methods could not be assigned to any chromosomal location (Figure 3A). Nine types of circRNAs were categorized, out of which exon–exon and intergenic–intergenic types, respectively, constitute the major share of ∼69% (#4,381) and ∼24% (#1,223) with the traditional RNA-Seq method (Figure 3B) and ∼40% (#28,248) and ∼56% (#39,647) by the i-tdMDA-NGS method (Figure 3B). The maximum number of circRNA, ∼95% (#6,040) from traditional RNA-Seq and ∼93.5% (#66,331) from i-tdMDA-NGS falls in sizes <1000 nt (Figure 3C). ∼27% (#1,728) and 5.2% (#3,715) of identified circRNA parental genes produced single circRNA, whereas ∼14 and ∼8% of the identified genes were observed to produce >1 circRNAs through the traditional RNA-Seq and i-tdMDA-NGS methods, respectively (Figure 3F). However, ∼5% (#315) and ∼55% (#39,460) of total circRNAs identified spanned the unannotated sequences in both the traditional method and i-tdMDA-NGS, respectively (Figure 3F). Interestingly, a significant number of circRNAs (#1,728 in traditional RNA-Seq and #3,715 in i-tdMDA-NGS) were observed to match with multiple gene sequences as well (Figure 3F). Both the methods yielded (∼93–95)% of the circRNAs having non-canonical splice junctions (Figure 3E), whereas ∼6% (#390) and ∼1% (#64) of circRNAs were having GT/AG and CT/AC splice junctions, respectively, with traditional RNA-Seq (Figure 3E) as opposed to ∼3% of circRNAs in both categories when analyzed by i-tdMDA-NGS (Figure 3E). It is interesting to note that the expression of 26.3% (#1,675) and 14.5% (#10,313) of circRNAs is more than its linear counterpart when analyzed by traditional RNA-Seq and i-tdMDA-NGS, respectively (Figure 3F), mostly distributed between chromosomes 1 and 6.

**FIGURE 4:**
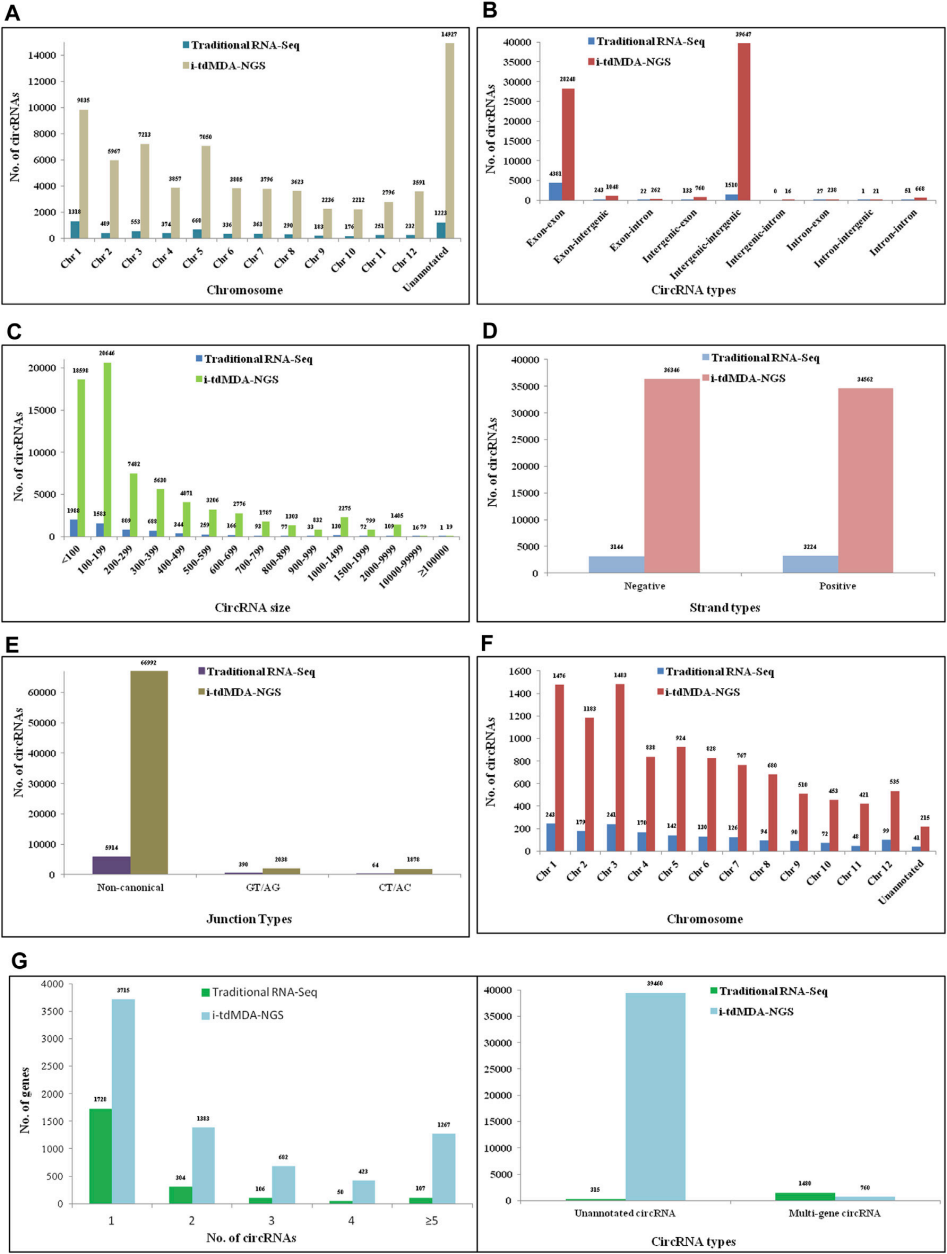
Comparative analysis of HeLa circRNAs by DCC. HeLa circRNAs identified by traditional circRNA-Seq and i-tdMDA-NGS were analyzed by the DCC computational pipeline and compared based on its **(A)** location on the chromosome, **(B)** types, **(C)** size, **(D)** strand location, and **(E)** junction types; **(F)** expression of circRNAs over the corresponding linear RNA across chromosomes and **(G)** number of circRNAs per host gene.

On the other hand, DCC analysis of HeLa RNA in the paired-dependent mode with the same number of processed reads gives 8,958 and 34,213 circRNAs from traditional RNA-Seq (analyzed in the stranded mode) and i-tdMDA-NGS (analyzed in the non-stranded mode), respectively (Figure 2), present across 22 pairs of autosomes, although 26.5% (#9,076) of i-tdMDA-NGS-derived circRNAs are coming from chromosome M (Figure 4A) and 14.7% (#1,314) are unannotated circRNAs in the case with the traditional RNA-Seq method (Figure 4A). The exon–exon type accounts for 70.2% (#6,290) of circRNAs, followed by exon-intergenic types at ∼11% (#999) when analyzed by the traditional RNA-Seq method (Figure 4B), whereas a majority are intergenic–intergenic types (∼63.8%) with i-tdMDA-NGS, followed by intron–intron types (16.85%) and exon–exon types, which constitutes only ∼10.7% of circRNAs (Figure 4B). The size of circRNAs stands >30% each for <1,000 nt, 1,000–9,999 nt, and ≥10,000 nt categories with the traditional RNA-Seq method (Figure 4C), whereas it is ∼11% (#3,744), ∼73.12% (#25,017), and ∼16% (#5,452) according to the i-tdMDA-NGS method (Figure 4C). The gene giving a single circRNA accounted for ∼26% (#2,318) and 9.6% (#3,292), the gene giving >1 circRNA covered 41.4% (#3,708) and 14.8% (#5,071). In addition, circRNAs arising from unannotated gene constituted ∼5% (#442) and 54.5% (#18,650) apart from 27.8% (#2,490) and 21% (#7,200) of circRNAs coming from multiple genes when analyzed by traditional RNA-Seq and i-tdMDA-NGS, respectively (Figure 4G). Using the traditional RNA-Seq method, DCC accounted for 52.27% (#4,682) and 47.19% (#4,227) of HeLa circRNAs with non-canonical and GT/AG canonical splice junctions, respectively (Figure 4E), whereas a significant 92.58% (#31,673) of circRNAs was formed by non-canonical splice junctions, followed by ∼3.7% of circRNAs formed by both GT/AG and CT/AC splice junctions each in the case of i-tdMDA-NGS (Figure 4E). Traditional RNA-Seq contributes ∼37 and ∼63% of HeLa circRNAs from negative and positive strands, respectively, whereas ∼50% of circRNAs are originating from both strands each in i-tdMDA-NGS (Figure 4D). Last, the expression of 11.5% (#3,933) to 12.7% (#1,139) of HeLa circRNAs was more than that of their corresponding linear mRNAs when analyzed by both the methods (Figure 4F).

Moreover, rice RNA processed reads from both the methods were also analyzed in the paired-independent plus stranded mode using the DCC computational pipeline. In this case, the total number of identified circRNAs increased to 17,509 and 179,783 from traditional RNA-Seq and i-tdMDA-NGS, respectively (Figure 2), equally distributed across both strands (Supplementary Figure S8D), and their locations were undetermined (∼13–14%) and mostly concentrated on chromosomes 1, 2, 3, or 5, accounting for 35–38% of total circRNAs (Supplementary Figure S8A). Traditional RNA-Seq constituted 61.72% (#10,807) and 28.68% (5,021) of exon–exon and intergenic–intergenic circRNAs, respectively (Supplementary Figure S8B), which was found to be the opposite phenomenon (31.81 and 62.09%) when analyzed by i-tdMDA-NGS (Supplementary Figure S8B). Although circRNAs were classified under lncRNA, a considerable number were <100 nt (∼33% - i-tdMDA-NGS to >43% - traditional RNA-Seq). A majority of circRNAs identified by traditional RNA-Seq (53.7%) and i-tdMDA-NGS (61.9%) were between 100 and 999 nt, whereas the remaining were ≥1000 nt (Supplementary Figure S8C). Most of the circRNAs were formed by non-canonical splice junctions (>95%), followed by circRNAs with GT/AG + CT/AC combinations together (Supplementary Figure S8E). About 33.17% (#5,807) of circRNAs were having expressions more than the linear counterparts when the data were analyzed by traditional RNA-Seq as compared to 22.5% (#40,396) analyzed by i-tdMDA-NGS (Supplementary Figure S8F). ∼28% (#4,936) and a significant ∼62% (#111,306) circRNAs were arising from unannotated genes; single circRNA-producing genes covered only 11.46% (#2,206) and 1.5% (#2,708) of all the identified circRNA parental genes, whereas >1 circRNAs backspliced out from a single gene constituted ∼24% (#2,294) and ∼6% (#6,998) of the total circRNA gene pool identified upon analysis through traditional RNA-Seq and i-tdMDA-NGS, respectively (Supplementary Figure S8G).

### CircRNA Validation

**FIGURE 5:**
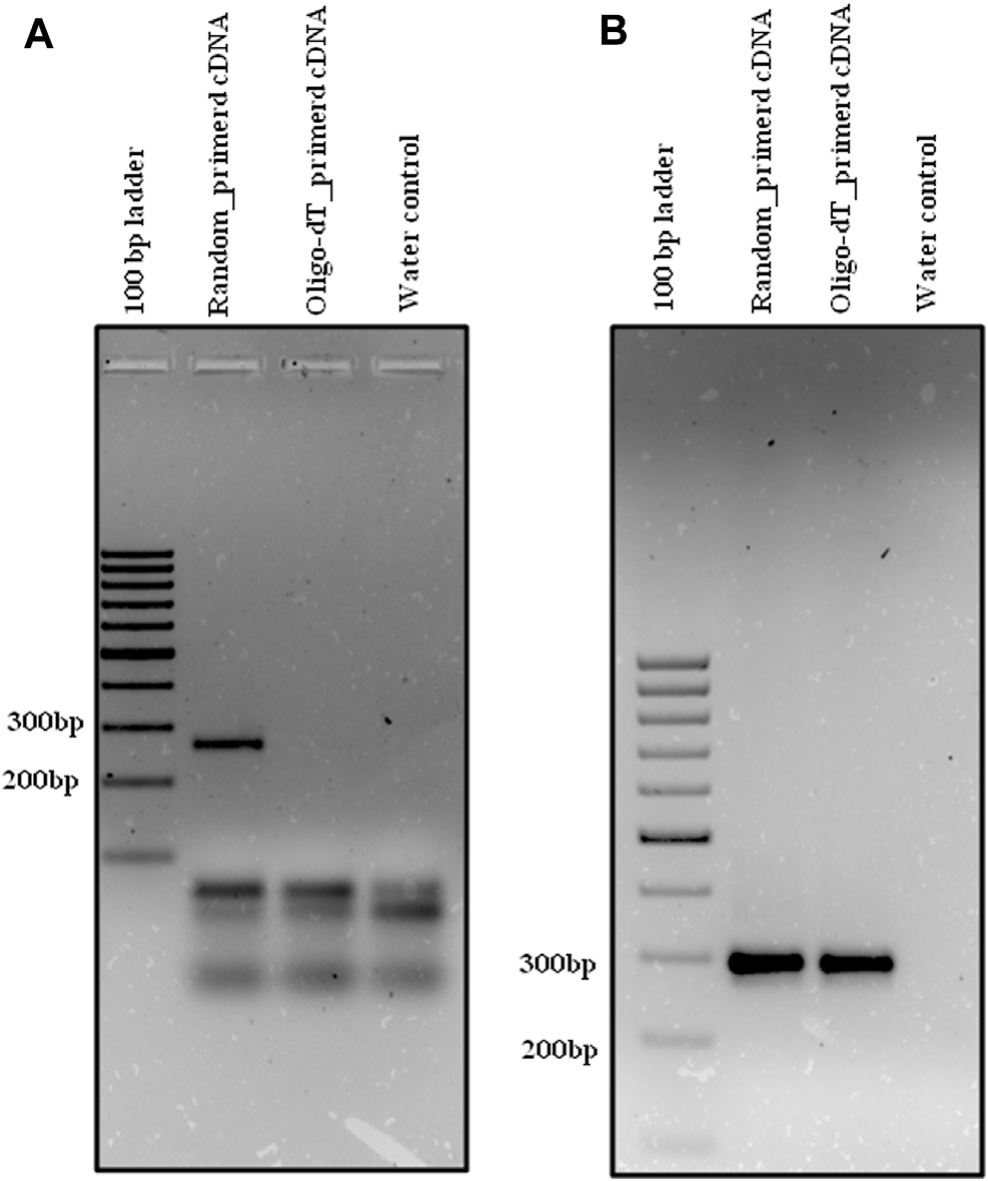
Divergent PCR in rice. Divergent PCR using **(A)** osi_DC_02 yielded ∼218 bp amplicon with random-primed cDNA but not with oligo-dT-derived cDNA, whereas convergent PCR using **(B)** os_β-actin yielded ∼126 bp amplicon with both random-primed cDNA and oligo-dT-derived cDNA. Generuler 100 bp ladder was loaded as a marker.

Primers were designed for randomly selected circRNA candidates from both rice and HeLa cell lines for the validation. Both random primed and oligo-dT derived cDNA were synthesized using DNase-treated RNA from both rice and HeLa cell lines as mentioned earlier in *Enrichment of circRNA and its Reverse Transcription*. The divergent primer, Osi_DC_02 (Table 1), was designed for validation of rice circRNA, osi_circ_2–187437-187904_(-), identified by i-tdMDA-NGS in combination with the paired-independent stranded mode analysis by DCC. Divergent PCR with the osi_DC_02 primer at T_A_-55°C showed an expected amplicon of ∼218 bp with random-primed cDNA but not with oligo-dT-derived cDNA (Figure 5A), whereas convergent PCR with the rice-specific *β*-actin primer (Table 2) at T_A_-55°C gave ∼126 bp expected amplicon with both random-primed and oligo-dT-derived cDNA (Figure 5B). This ∼218 bp amplicon was cloned in pGEMT-Easy vector, confirmed by *EcoR*I digestion and sequenced with the universal T7 forward primer, which revealed the exact matching of nucleotide sequences including the junction point sequence (AC/GT) with an additional G nt at its junction when compared with the circRNA, osi_circ_2-187437-187904_(-) (Supplementary Figure S9). However, there was a Ct value while using both types of cDNA upon analyzing the rice qRT-PCR data (Supplementary Figures S10A,B). As expected, a ∼218 bp circRNA-specific amplicon was produced with random-primed cDNA only (using the osi_DC_02 primer), whereas an ∼126 bp *β*-actin-specific qRT-PCR product was amplified from both random-primed and oligo-dT derived cDNA (Supplementary Figures S10C–E). Careful gel analysis revealed the additional presence of primer dimer amplification with every sample tested in triplicates using the osi_DC_02 primer (Supplementary Figures S10C–E), which was further supported by an additional peak in the dissociation curve but not with the rice-specific *β*-actin primer, which explains the possible reason behind Ct values of all samples (Supplementary Figures S10F–H).

**TABLE 2:** Table showing the list of convergent primers used in the study.

Organism	Housekeeping Gene	Primer	Sequence (5′-3′)	Length (nt)	Amplicon Size (bp)
Rice	Rice-specific *β*-actin	Os_β-actin FOR	CTT​GCT​GGG​CGT​GAT​CTC​A	19	126
		Os_ *β*-actin REV	CAG​GGC​GAT​GTA​GGA​AAG​CT	20	
HeLa cell lines	HeLa-specific *β*-actin	Hl_β-actin FOR	TGG​ACT​TCG​AGC​AAG​AGA​TG	20	292
		Hl_β-actin REV	GTG​ATC​TCC​TTC​TGC​ATC​CTG	21	

**FIGURE 6:**
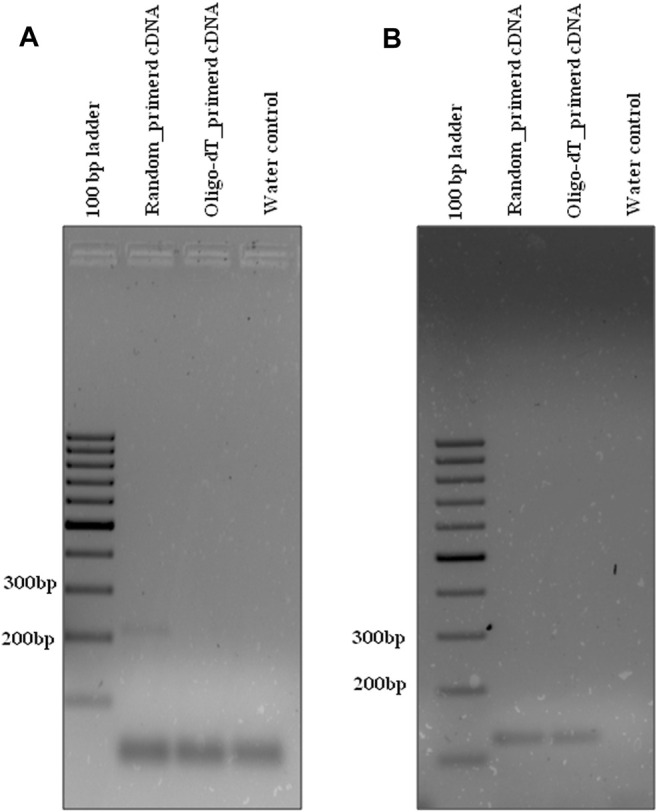
Divergent PCR in HeLa cell lines. Divergent PCR using **(A)** hl_DC_02 yielded ∼267 bp amplicon with random-primed cDNA but not with oligo-dT-derived cDNA, whereas convergent PCR using **(B)** hl_β-actin yielded ∼292 bp amplicon with both random-primed cDNA and oligo-dT-derived cDNA. Generuler 100 bp ladder was loaded as a marker.

Similarly, the HL_DC_02 divergent primer (Table 1) was designed for validation of circRNA, hl_circ_19-8963380-8964261_(-), identified by i-tdMDA-NGS and analyzed by the paired-dependent non-stranded mode from HeLa cell lines using the DCC computational pipeline. As expected, divergent PCR using the HL_DC_02 primer at T_A_-45°C yielded the anticipated ∼267 bp amplicon with random-primed cDNA but not with oligo-dT-derived cDNA (Figure 6A) as opposed to ∼292 bp PCR amplicon derived from the human-specific *β*-actin primer (T_A_-55°C) (Table 2) from both types of cDNAs (Figure 6B). The divergent PCR product was gel-eluted and Sanger-sequenced with the circRNA-specific forward primer, which matched with the i-tdMDA-NGS-derived circRNA, hl_circ_19–8963380-8964261_(-), sequence including the junction sequence (CC/CG) with an additional incorporation of T nt at its junction (Supplementary Figure S11). Moreover, gel analysis of qRT-PCR data also showed the specific ∼267 bp amplicon in random-primed cDNA only with the HL_DC_02 primer and the ∼292 bp amplicon in both random-primed and oligo-dT derived cDNA (with human-specific *β*-actin primer) apart from primer dimer amplification in triplicates (Supplementary Figures S12C–E), which explains the presence of an additional peak in the dissociation curve (Supplementary Figures S12F–H) and a corresponding Ct value for each sample (Supplementary Figures S12A,B).

## Discussion

CircRNA research has grown manifold in the past decade. Recently, a lot of research is focused on functional aspects of circRNAs and the role in biomarker development (Zhang S. et al., 2018; Verduci et al., 2021). However, identification of circRNAs is an intrinsic and fundamental requirement before exploring and validating the numerous putative functions. Apart from microarrays (Zhang Z. et al., 2018; Li et al., 2019; Li et al., 2021; Zhong et al., 2021), circRNA ampli-seq panels, fluorescent padlock probes (Zaghlool et al., 2018) or the RNase H cleavage-based assay (Barrett and Salzman, 2016), and a few other methods require prior circRNA sequence information; dependency on NGS-based approaches is found to be the only viable option for *de novo* circRNA identification till date (Gao et al., 2015). This, which we termed as traditional RNA-Seq here, could be performed either by sequencing of total RNA or by poly(A)-depleted RNA or/and linear RNA-depleted RNA (Xiao and Wilusz, 2019) or rRNA-depleted RNA (Memczak et al., 2012; Salzman et al., 2012; Westholm et al., 2014; Gao et al., 2015) or combining two or all the approaches together (Pandey et al., 2019; Shao et al., 2019; Han et al., 2020; Guria et al., 2021). However, these strategies require a huge amount of NGS reads for detection of a handful of circRNAs (Jeck and Sharpless, 2014) as previously evidenced in the literature (Salzman et al., 2013). Moreover, the usage of RNA for/in circRNA-Seq limits its stability, which often results in sample degradation during transport. On the other hand, bioinformatic pipelines are a rate-limiting step in circRNA detection since it is found that usage of different circRNA pipelines for analysis of the same raw data results in circRNA identification discrepancy (Hansen et al., 2016; Szabo and Salzman, 2016; Zeng et al., 2017; Hansen, 2018). CircRNA identification by these approaches may have an under-representation of its actual number present in an organism because of exclusion of abysmally expressed circRNAs and/or the presence of false positives.

Here, in this article, we have introduced a new method of circRNA identification which is actually a refined version of our previously published method (Guria et al., 2019). The concept of td-MDA was utilized as an additive inclusion prior to NGS for circRNA detection. However, phi29 DNA polymerase-mediated MDA using ERRH is prone to TIA (Wang W. et al., 2017). Hence, different concentrations of ERRH and ERRP were tested at varied incubation temperatures and times. As expected, ERRP was proved better over ERRH in TIA elimination since the presence of the C18 spacer at the 5′end of ERRP is assumed to inhibit polymerase jumping and self-priming (Wang W. et al., 2017). On the other hand, DNase-treated RNAs from both Indica rice and HeLa cell lines were treated for rRNA depletion, followed by leftover linear RNA removal by RNase R. The enriched RNA, containing mostly putative circRNAs, was converted into long RNA:DNA hybrids using RT having a mutation in the RNase H domain. The advantage of H minus RT over normal RT is that the enzyme does not degrade RNA in the RNA-DNA hybrid; during the synthesis of the first strand, cDNA results in obtaining full-length cDNAs from long templates. Thus, in order to degrade RNA from RNA-DNA hybrids, RNase H treatment was followed to generate long ss cDNA, which was used as a template for phi29 DNA polymerase-mediated td-MDA using an optimized 30 µM final concentration of ERRP at 28°C for 21 h, followed by sequencing to generate 10 million PE reads/sample. For comparative analysis, the treated RNA was also utilized for NGS at the same sequencing reads by a conventional way. Further, NGS reads from both the current methods and the earlier method, tdMDA-NGS (Guria et al., 2019), were analyzed for circRNA identification using both DCC and CIRI2 circRNA pipelines. It is inferred that i-tdMDA-NGS is better over traditional RNA-Seq in yielding the maximum number of circRNAs when analyzed using DCC from both rice and HeLa cells. The result is the same using CIRI2 for rice but unexpectedly the opposite for HeLa cells due to unknown reasons. CIRI2 yielded fewer circRNAs than DCC for either i-tdMDA-NGS or traditional RNA-Seq because of identification of circRNAs with only canonical splice junctions. It is equally interesting to find out that i-tdMDA-NGS is comparatively better than tdMDA-NGS (Guria et al., 2019) in yielding more circRNAs when analyzed by DCC and CIRI2 pipelines in rice (Supplementary Figures S13, S14). Further, the functional characterization of rice-validated circRNA can be analyzed by making over-expression constructs (Sharma et al., 2021). Moreover, analysis of i-tdMDA-NGS and traditional RNA-Seq data by the DCC paired-independent stranded mode showed a >2.5-fold spike in detection over DCC paired-dependent mode analysis. Overall, our new method is much better in identifying more circRNAs than tdMDA-NGS (Guria et al., 2019) and traditional RNA-Seq either at the same sequencing depth or as mentioned in the literature previously (Jakobi et al., 2016; Wang Y. et al., 2017; Wang et al., 2018), which ultimately reduced the cost of circRNA identification significantly.

## Conclusion

We formulated a new method, i-tdMDA-NGS, for circRNA identification which is superior over the current traditional circRNA-Seq in detecting a large number of circRNAs at a much-reduced cost. However, we aim to validate and authenticate the existence of a larger number of circRNAs identified by our method in near future by including other experiments such as Northern blotting, the RNase protection assay, and so forth.

## Data Availability

The datasets presented in this study can be found in online repositories. The names of the repository/repositories and accession number(s) can be found in the article/Supplementary Material.
