# Supplementary figures and images for: Cost-Effective Transcriptome-Wide Profiling of Circular RNAs by the Improved-tdMDA-NGS Method
Source: Front Mol Biosci. 2022 May 13;9:886366. doi: 10.3389/fmolb.2022.886366 (PMC9136142; doi:10.3389/fmolb.2022.886366)

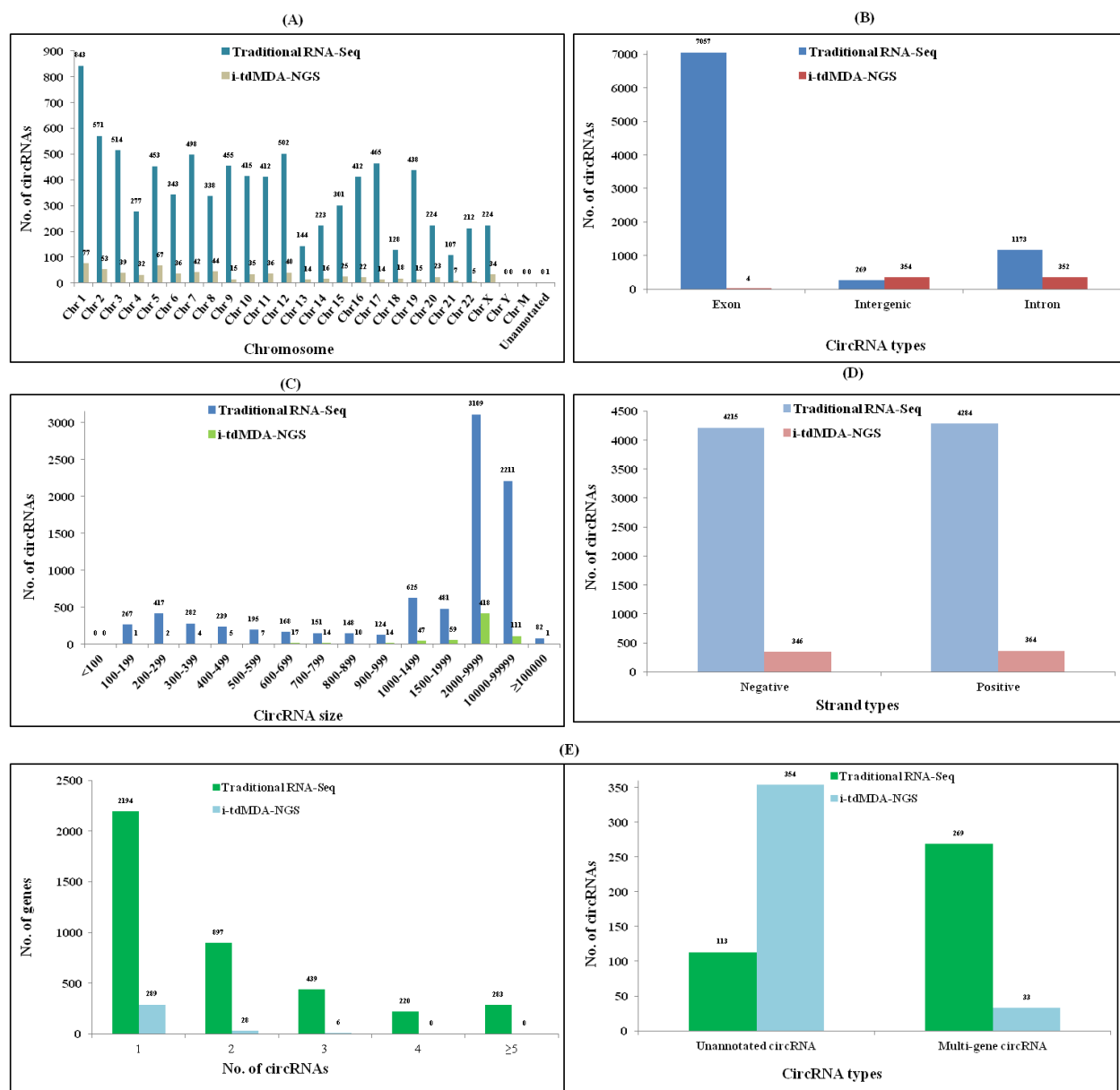

Supplement: Supplementary file 1 [file DataSheet7.pdf]

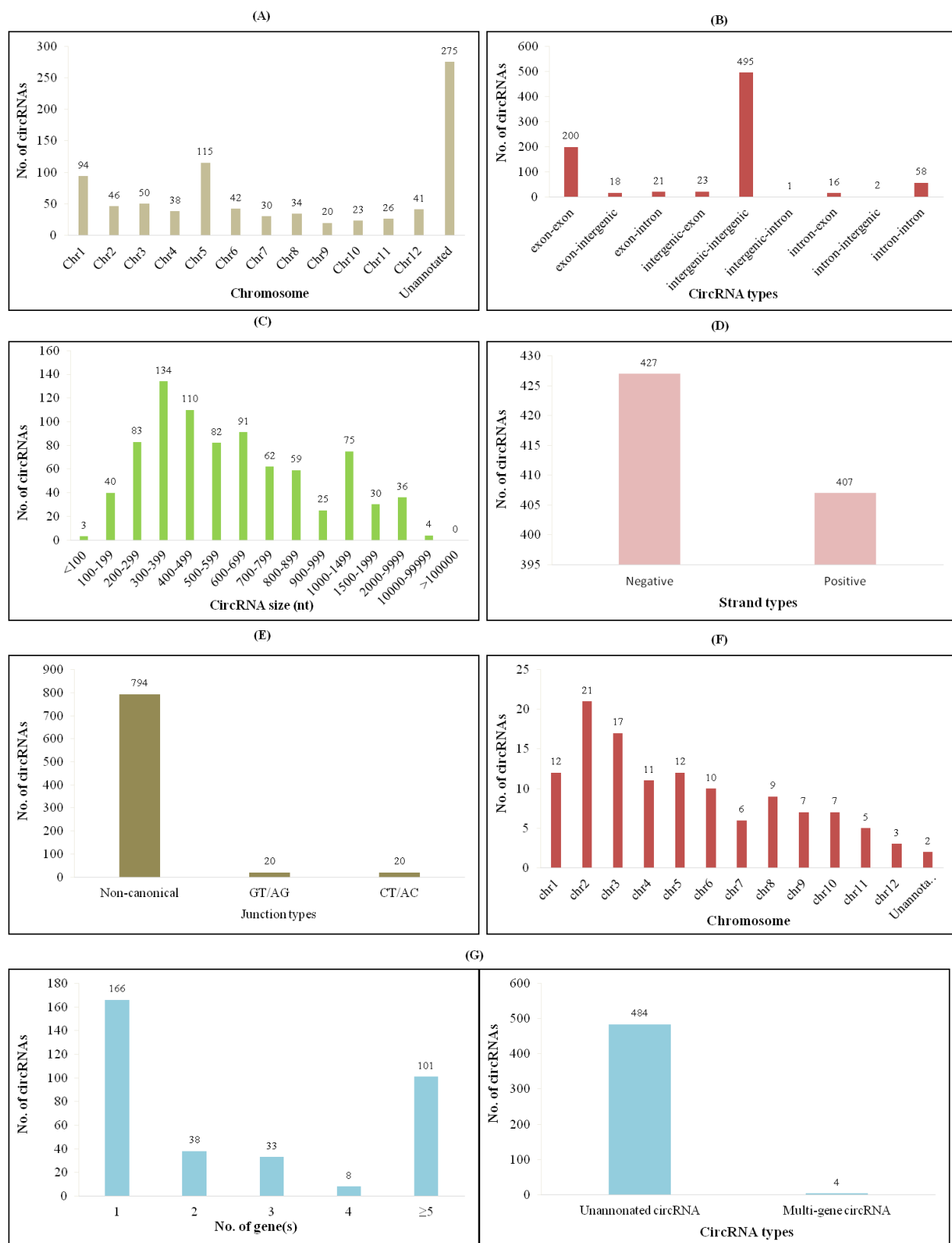

Supplement: Supplementary file 2 [file DataSheet13.PDF]

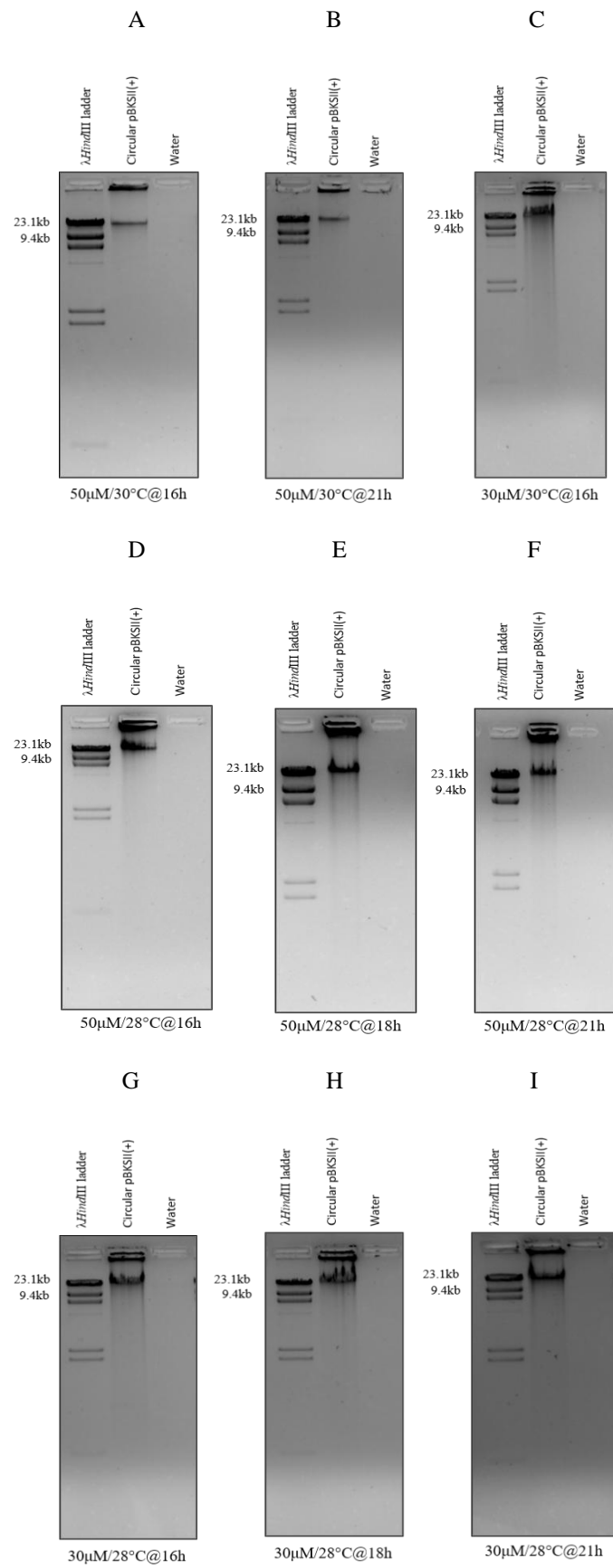

Supplement: Supplementary file 3 [file DataSheet2.PDF]

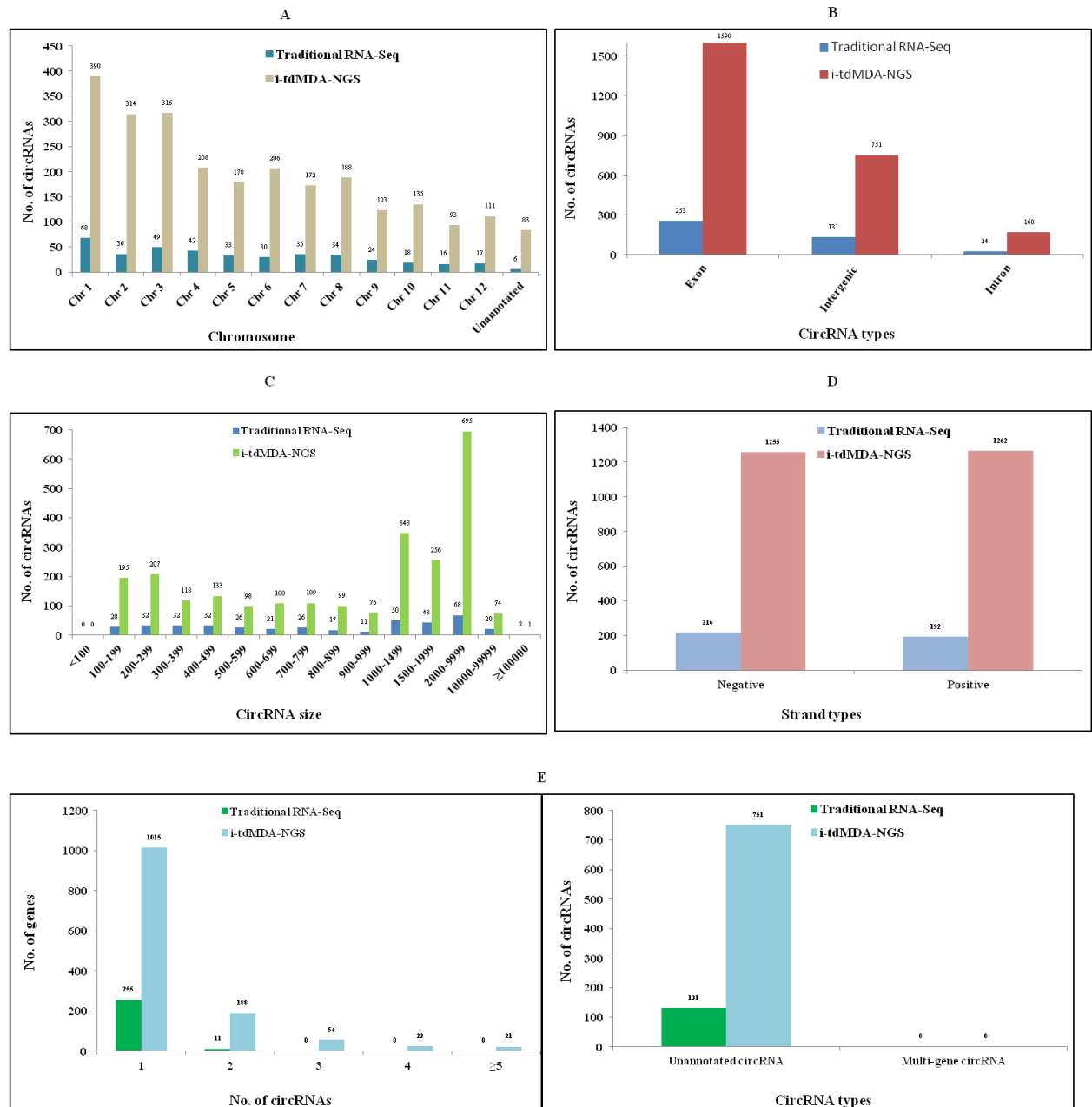

Supplement: Supplementary file 5 [file DataSheet6.pdf]

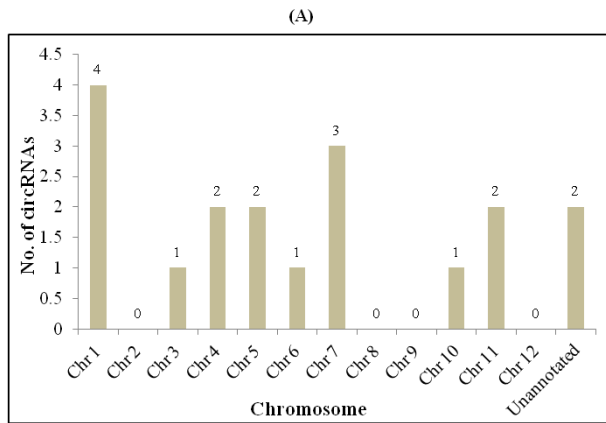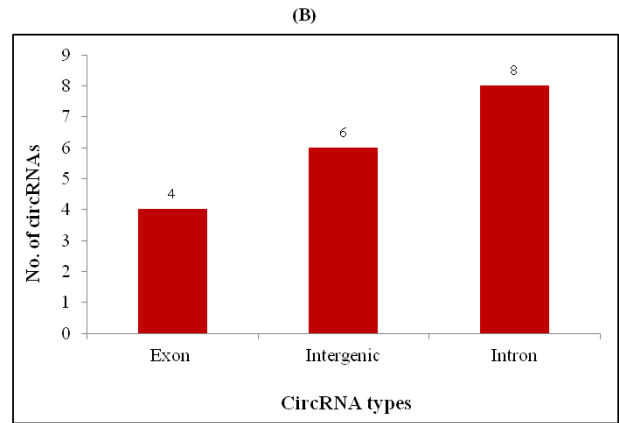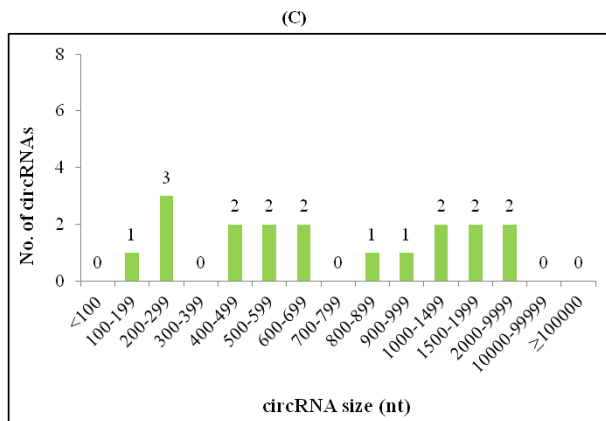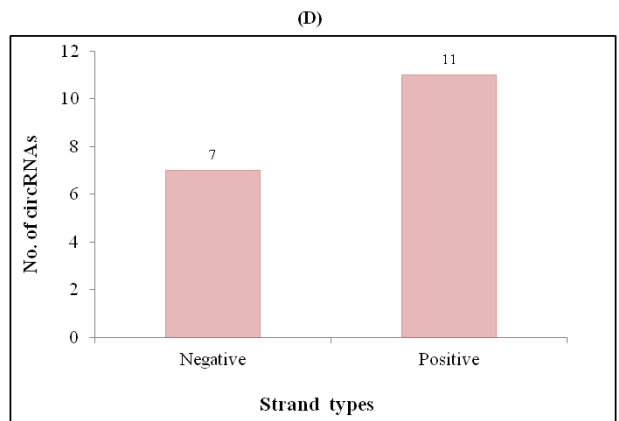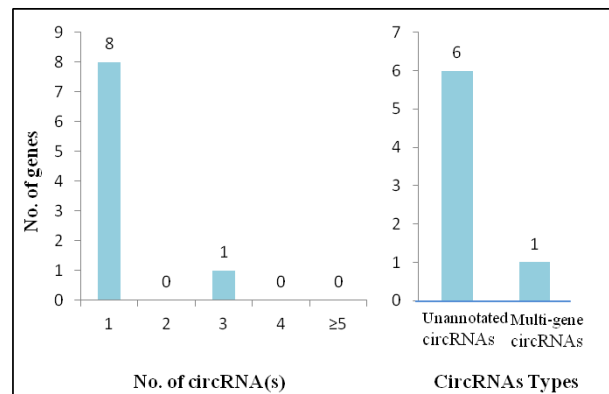

Supplement: Supplementary file 6 [file DataSheet14.PDF]

A

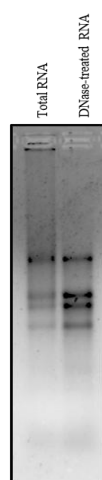

Indica Rice RNA

B

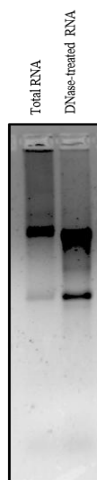

HeLa cell lines RNA

Supplement: Supplementary file 9 [file DataSheet3.PDF]

A

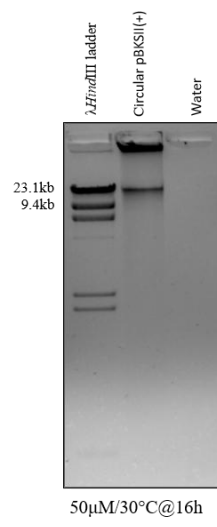

B

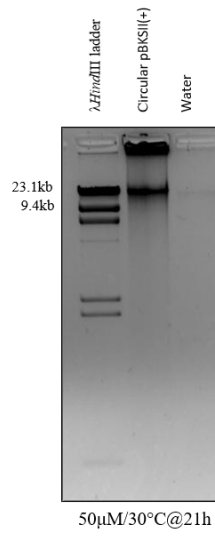

C

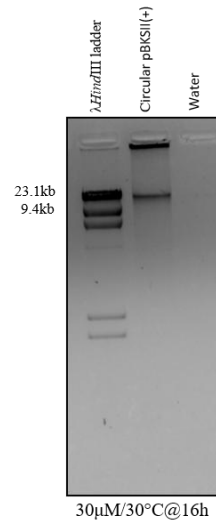

Supplement: Supplementary file 10 [file DataSheet1.PDF]

A

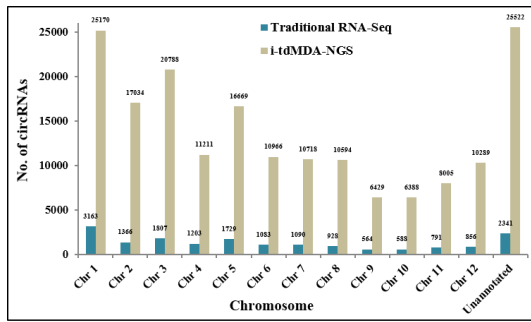

B

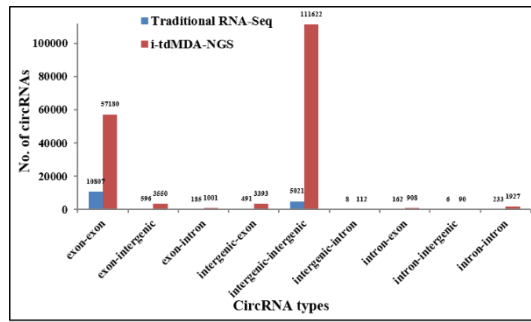

C

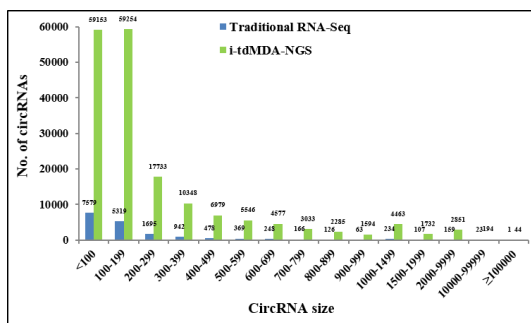

D

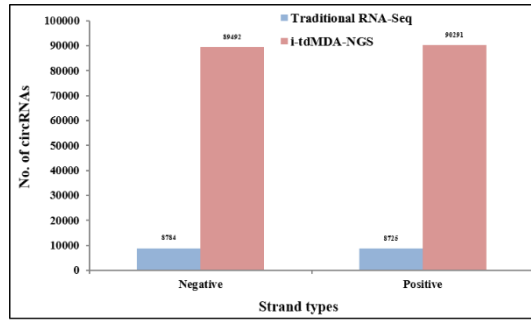

E

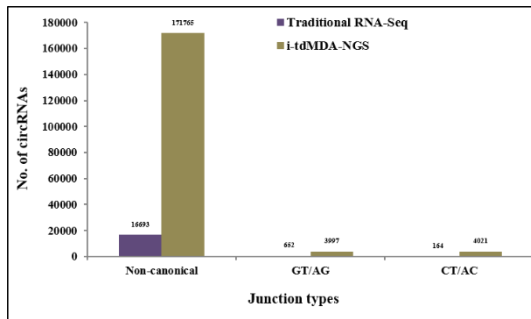

F

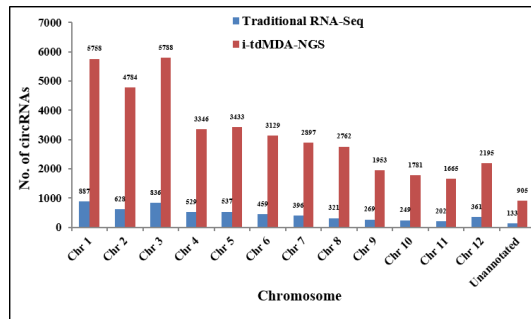

G

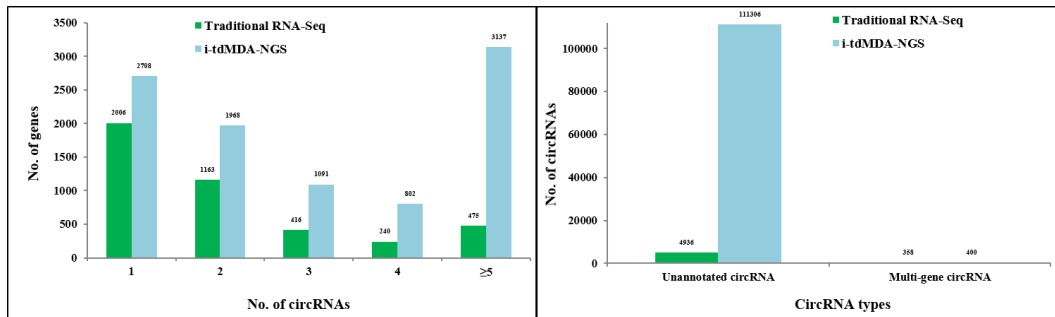

Supplement: Supplementary file 13 [file DataSheet8.pdf]
