# Supplementary material for: Cost-Effective Transcriptome-Wide Profiling of Circular RNAs by the Improved-tdMDA-NGS Method
Source: Front Mol Biosci. 2022 May 13;9:886366. doi: 10.3389/fmolb.2022.886366 (PMC9136142; doi:10.3389/fmolb.2022.886366)
Supplement: Supplementary file 4 [file DataSheet4.pdf]

Traditional RNA-Seq	Indica (PB-1) rice
Raw reads	34,299,487
Processed @ phred25	31,261,868 (91.14%)

itd-MDA-NGS	Indica (PB-1) rice
Raw reads	38,618,983
Processed @ phred25	36,086,992 (93.44%)

tdMDA-NGS (Guria et al., 2019)	Indica (PB-1) rice
Raw reads	34,877,691
Processed @ phred25	29,813,764 (85.48 %)
