# Supplementary material for: Cost-Effective Transcriptome-Wide Profiling of Circular RNAs by the Improved-tdMDA-NGS Method
Source: Front Mol Biosci. 2022 May 13;9:886366. doi: 10.3389/fmolb.2022.886366 (PMC9136142; doi:10.3389/fmolb.2022.886366)
Supplement: Supplementary file 7 [file DataSheet9.pdf]

NGS-derived circRNA (Osi_circ_2-187437-187904_(-)) sequence

GTGACCTGAAGCCTGGTTCGGACGTGAAGATCACGGGGCCAGTGGGGAAGGAGATGCTGATGCCCAA
GGACCCCAACGCCACCATCATCATGCTGGGCACCGGCACCGGCATCGCGCCCTTCAGGTCCTTCCTGT
GGAAGATGTTCTTCGAGGAGCACGACGACTACAGGTTCAACGGCCTGGCGTGGCTCTTCCTCGGGGTG
CCCACCAGCAGCACGCTGCTGTACAGGGAGGAGTTCGAGCGGATGAAGGAGATCGCGCCGGAGAGGT
TCCGGCTGGACTTCGCGGTGAGCCGGGAGCAGACGAACGCGGCGGGGGAGAAGATGTACATCCAGAC
GCGGATGGCGGAGTACAAGGACGAGCTGTGGGAGCTGCTCAAGAAGGACAACACCTACGTCTACATG
TGCGGCCTCAAGGGCATGGAGAAAGGCATCGACGACATCATGATCGACCTCGCTGCAAAAGAC

Sangar sequencing result of Osi_DC_02-derived circRNA (Osi_circ_2-187437-187904_(-)) sequence using Universal T7 forward primer

CCGCCATGGCGGCCGCGGGAATTCGATTGGCATCGACGACATCATGATCGACCTCGCTGCAAAAGA
CGGTGACCTGAAGCCTGGTTCGGACGTGAAGATCACGGGGCCAGTGGGGAAGGAGATGCTGATGCCC
AAGGACCCCAACGCCACCATCATCATGCTGGGCACCGGCACCGGCATCGCGCCCTTCAGGTCCTTCCT
GTGGAAGATGTTCTTCGAGGA**GCACGACGACTACAGG**AATCACTAGTGAATTCGCGGCCGCCTGCAG
GTCGACCATATGGGAGAGCTCCCAACGCGTTGGATGCATAGCTTGAGTATTCTATAGTGTCACCTAAA
TAGCTTGGCGTAATCATGGTCATAGCTGTTTCCTGTGTGAAATTGTTATCCGCTCACAATTCCACACAA
CATATGAGCCGGAAGCATAAAGTGTAAAGCCTTGGGGTGCCTAATGAGTGAGCTAACTCACATTAATT
GCGTTGCGCTTACTGCCCGCTTTTCT

Osi_DC_02 forward primer (Highlighted is the region of primer matched in Sangar sequencing)

AGAAAGGCATCGACGACATC

Osi_DC_02 reverse primer (Highlighted is the region of primer matched in Sangar sequencing)

GCACGACGACTACAGGTTCA

NGS-derived circRNA splice junction – AC/GT

CircRNA splice junction from sanger sequencing – ACGGT (one extra G)
