# Supplementary material for: Cost-Effective Transcriptome-Wide Profiling of Circular RNAs by the Improved-tdMDA-NGS Method
Source: Front Mol Biosci. 2022 May 13;9:886366. doi: 10.3389/fmolb.2022.886366 (PMC9136142; doi:10.3389/fmolb.2022.886366)
Supplement: Supplementary file 8 [file DataSheet11.pdf]

NGS-derived circRNA (Hl_circ_19-8963380-8964261_(-)) sequence

CGCCTTCCAATATTTTGTTGACATCACAAGGGCACAGTCCCTCCTCTACTCCACCTGTGACCTCAGT
TTTCTTGTCTGAGACCTCTGGCCTGGGGAAGACCACAGACATGTCGAGGATAAGCTTGGAACCTGG
CACAAGTTTACCTCCCAATTTGAGCAGTACAGCAGGTGAGGCGTTATCCACTTATGAAGCCTCCAG
AGATACAAAGGCAATTCATCATTCTGCAGACACAGCAGTGACGAATATGGAGGCAACCAGTTCTG
AATATTCTCCTATCCCAGGCCATACAAAGCCATCCAAAGCCACATCTCCATTGGTTACCTCCCACA
TCATGGGGGACATCACTTCTTCCACATCAGTATTTGGCTCCTCCGAGACCACAGAGATTGAGACAG
TGTCCTCTGTGAACCAGGGACTTCAGGAGAGAAGCACATCCCAGGTGGCCAGCTCTGCTACAGAG
ACAAGCACTGTCATTACCCATGTGTCTAGTGGTGATGCTACTACTCATGTCACCAAGACACAAGCC
ACTTTCTCTAGCGGAACATCCATCTCAAGCCCTCATCAGTTTATAACTTCTACCAACACATTTACAG
ATGTGAGCACCAACCCCTCCACCTCTCTGATAATGACAGAATCTTCAGGAGTGACCATCACCACCC
AAACAGGTCCTACTGGAGCTGCAACACAGGGTCCATATCTCTTGGACACATCAACCATGCCTTACT
TGACAGAGACTCCATTAGCTGTGACTCCAGATTTTATGCAATCAGAGAAGACCACTCTCATAAGCA
AAGGTCCCAAGGATGTGTCCTGGACAAGCCCTCCCTCTGTGGCAGAAACCAGCTATCCCTCTTCCC
TGACACCTTTCTTGGTCACAACC

Sangar sequencing result of Hl_DC_02-derived circRNA (Hl_circ_19-8963380-8964261_(-)) sequence using Hl_DC_02 forward primer

CTTCCTCTTCCCTGGTACCTATCCATGCTACACCTCGCCTTCCAATATTTTGTTGACATCACAAGGG
CACAGTCCCTCCTCTACTCCACCTGTGACCTCAGTTTTCTTGTCTGAGACCTCTGGCCTGGGGAAGA
CCACAGACATGTCGAGGATAAGCTTGGAACCTGGCACAAGTTTACCTCCCAATTTGAGCAGTACA
GCAGGTGAGGCGTTATC

Hl_DC_02 forward primer

GTCCCAAGGATGTGTCCTGG

Hl_DC_02 reverse primer (Highlighted is the region of primer matched in Sangar sequencing)

GATAACGCCTCACCTGCTGT

NGS-derived circRNA splice junction – CC/CG

CircRNA splice junction from sanger sequencing – CCTCG (one extra T)
