# Supplementary material for: Cost-Effective Transcriptome-Wide Profiling of Circular RNAs by the Improved-tdMDA-NGS Method
Source: Front Mol Biosci. 2022 May 13;9:886366. doi: 10.3389/fmolb.2022.886366 (PMC9136142; doi:10.3389/fmolb.2022.886366)
Supplement: Supplementary file 11 [file DataSheet5.pdf]

Traditional RNA-Seq	Hela cell lines
Raw reads	48,688,532
Processed @ phred25	48,599,461 (99.82%)

itd-MDA-NGS	Hela cell lines
Raw reads	29,834,900
Processed @ phred25	28,803,581 (96.54%)
