# Supplementary material for: Cost-Effective Transcriptome-Wide Profiling of Circular RNAs by the Improved-tdMDA-NGS Method
Source: Front Mol Biosci. 2022 May 13;9:886366. doi: 10.3389/fmolb.2022.886366 (PMC9136142; doi:10.3389/fmolb.2022.886366)
Supplement: Supplementary file 12 [file DataSheet12.pdf]

A

Hl_DC_02	Random primed cDNA (Ct)	Oligo-dT cDNA (Ct)
Test-1	30.016	36.668
Test-2	29.976	-
Test-3	29.773	35.984

B

Hl_β-actin	Random primed cDNA (Ct)	Oligo-dT cDNA (Ct)
Test-1	16.222	18.298
Test-2	16.138	18.296
Test-3	16.236	18.172

C

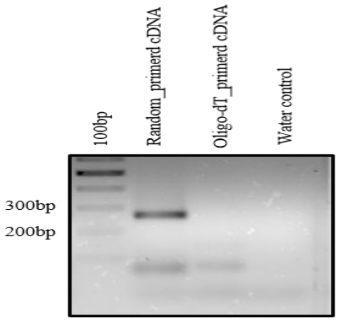

Hl_DC_02 (Test-1)

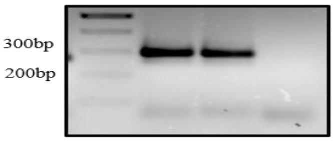

Hl_ β - actin (Test-1)

D

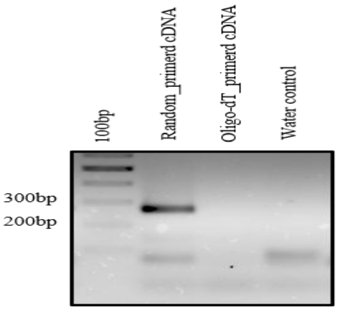

Hl_DC_02 (Test-2)

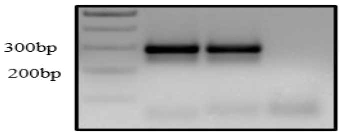

Hl_β-actin (Test-2)

E

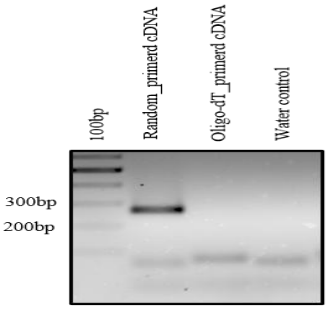

Hl_DC_02 (Test-3)

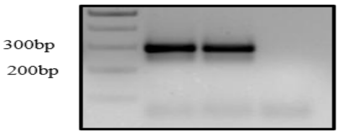

Hl_ β -actin (Test-3)

F

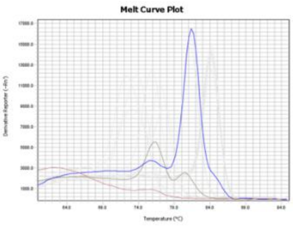

Hl_DC_02 (Test-1)

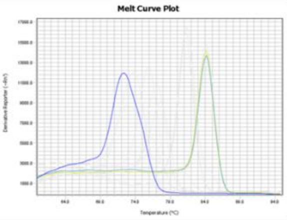

Hl_ β - actin (Test-1)

G

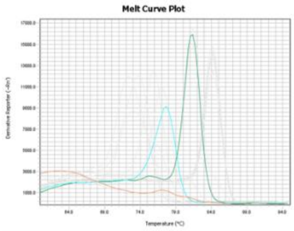

Hl_DC_02 (Test-2)

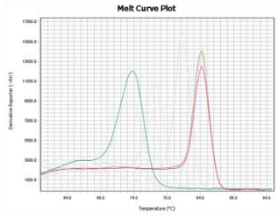

Hl_ β - actin (Test-2)

H

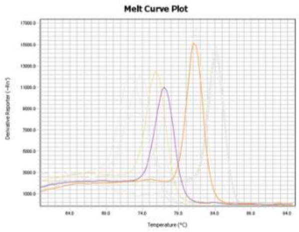

Hl_DC_02 (Test-3)

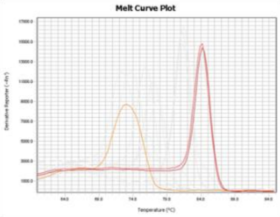

Hl_ β - actin (Test-3)
