# Supplementary material for: Cost-Effective Transcriptome-Wide Profiling of Circular RNAs by the Improved-tdMDA-NGS Method
Source: Front Mol Biosci. 2022 May 13;9:886366. doi: 10.3389/fmolb.2022.886366 (PMC9136142; doi:10.3389/fmolb.2022.886366)
Supplement: Supplementary file 14 [file DataSheet10.pdf]

A

Osi_DC_02	Random primed cDNA (Ct)	Oligo-dT cDNA (Ct)
Test-1	28.822	29.467
Test-2	28.867	29.288
Test-3	28.69	29.415

B

Os_β-actin	Random primed cDNA (Ct)	Oligo-dT cDNA (Ct)
Test-1	31.587	32.628
Test-2	31.938	32.718
Test-3	31.991	32.797

C

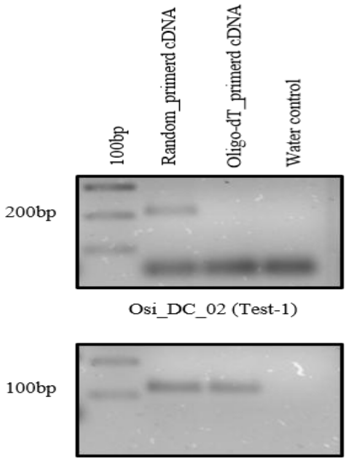

Osi_DC_02 (Test-1)

Os_β-actin (Test-1)

D

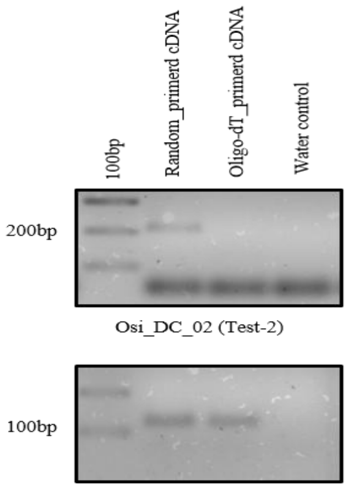

Osi_DC_02 (Test-2)

Os_β-actin (Test-2)

E

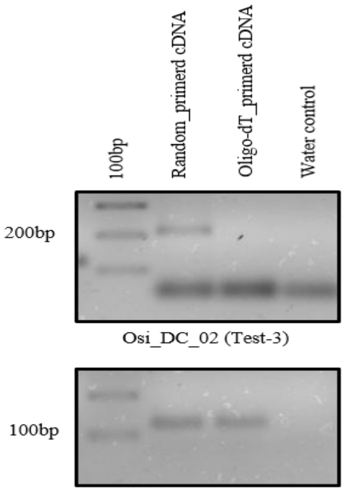

Osi_DC_02 (Test-3)

Os_β-actin (Test-3)

F

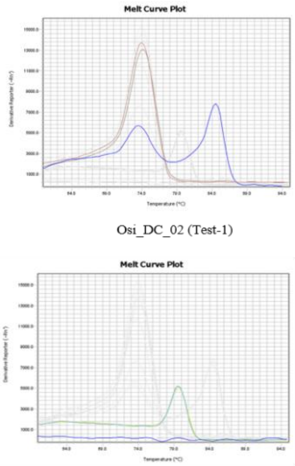

Osi_DC_02 (Test-1)

Os_β-actin (Test-1)

G

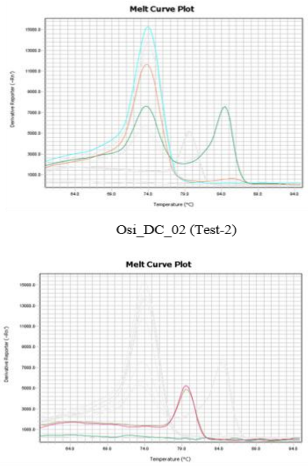

Osi_DC_02 (Test-2)

Os_β-actin (Test-2)

H

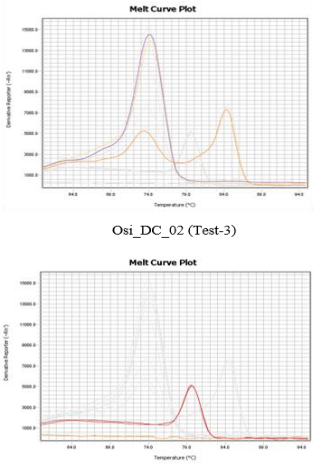

Osi_DC_02 (Test-3)

Os_β-actin (Test-3)
